# TUBERCULOSIS OF SPLEEN PRESENTING WITH PYREXIA OF UNKNOWN ORIGIN IN A NON-IMMUNOCOMPROMISED WOMAN

**DOI:** 10.4103/0970-2113.44134

**Published:** 2008

**Authors:** Prem Parkash Gupta, Sanjay Fotedar, Dipti Agarwal, Pradeep Sansanwal

**Affiliations:** 1 Department of Respiratory Medicine, Postgraduate Institute of Medical Sciences, Rohtak., India; 2 Department of Physiology, Postgraduate Institute of Medical Sciences, Rohtak., India; 3 Department of Pathology, Postgraduate Institute of Medical Sciences, Rohtak., India

**Keywords:** Splenic tuberculosis, CT guided splenic biopsy, Non-immunocompromised patient

## Abstract

Splenic lesions due to tuberculosis are extremely rare in immunocompetent indi-viduals and delays in diagnosis are frequent. Here, we describe a 49-year-woman presenting with pyrexia-of-unknown origin with no evidence of any immunodefi-ciency. Computed tomography of the abdomen showed an enlarged spleen having multiple small focal hypodense lesions; the later were confirmed to be of tubercu-lous etiology on histopathological examination. She had favorable response with anti-tubercular chemotherapy. We report this case of tuberculosis spleen in an im-munocompetent individual for its rarity and to highlight the fact that these patients can be managed by medical treatment effectively.

## INTRODUCTION

According to WHO, 2 billion people, equal to a third of the world's total population are infected with tuberculous bacilli, and global tuberculosis incidence is still growing at 1% a year.^1^ Out of various extrapulmonary tuberculous entities, the splenic tuberculosis is extremely rare and delay in diagnosis is frequent. Few of the reasons for this undue delay being nonspecific clinical presentation, difficulties in confirming the diagnosis and prevalent concepts requiring the surgical intervention for confirmation of the disease and its subsequent treatment. Though, tuberculosis is not so rare an infectious disease leading to splenic enlargement without involving it, splenic tuberculosis is not frequently seen, and that too restricted largely too immunocompromised population.^2^

## CASE REPORT

A 49-year-woman, married, housewife presented at our Institute with the symptoms of fever of undetermined origin for preceding 3 months. The fever was of low grade and intermittent in nature. There was no history of cough / sputum / haemoptysis / breathlessness. She had no history of diabetes, hypertension, or any other significant disease. She received medical advice from various private practionars as well as at community health centre of her town but her fever persisted. No history of trauma could be elicited from the patient. There was no history of tuberculosis in the family.

On clinical examination, she had average built and good nutrition with stable vital signs. She had respiratory rate 18/min along with normal breath sounds over bilateral lung fields. She had enlarged spleen that was tender on clinical examination. The liver was not palpable and the examination of other systems was not remarkable.

Her hemoglobin level was 13.6 g/dl; TLC was 10200/ mm^3^. Additional biochemical parameters including liver functions, blood sugar, blood urea, and serum creatine were with in normal limits. The patient was found to be human immunodeficiency virus (HIV) seronegative by enzyme-linked immunosorbent assay (ELISA). Her chest skiagrams were within normal limits. Tuberculin test was positive (an induration of 18 mm at 48 hours with 1 TU PPD). Induced sputum smears were negative for acid-fast bacilli (AFB).

**Figure 1:**
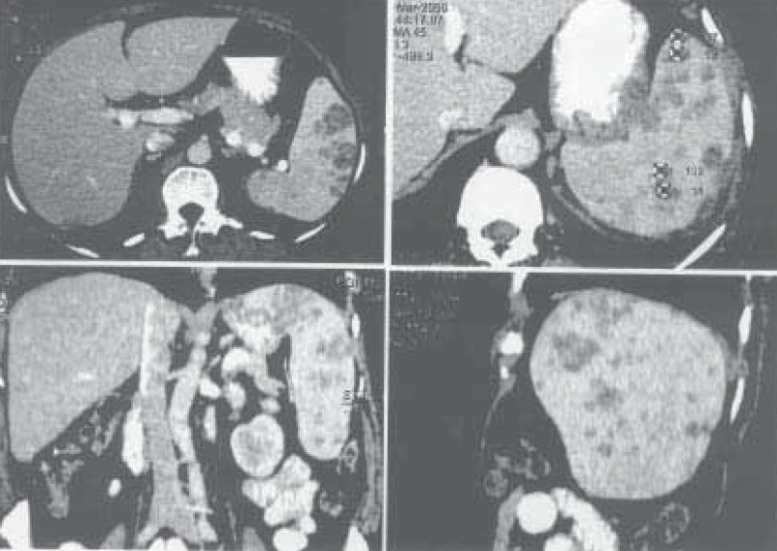
Computed tomogram of abdomen (A normal view, B enlarged view, C coronal section, and D sagital section) showing multiple small focal hypodense lesions that were suggestive of abscesses.

**Figure 2:**
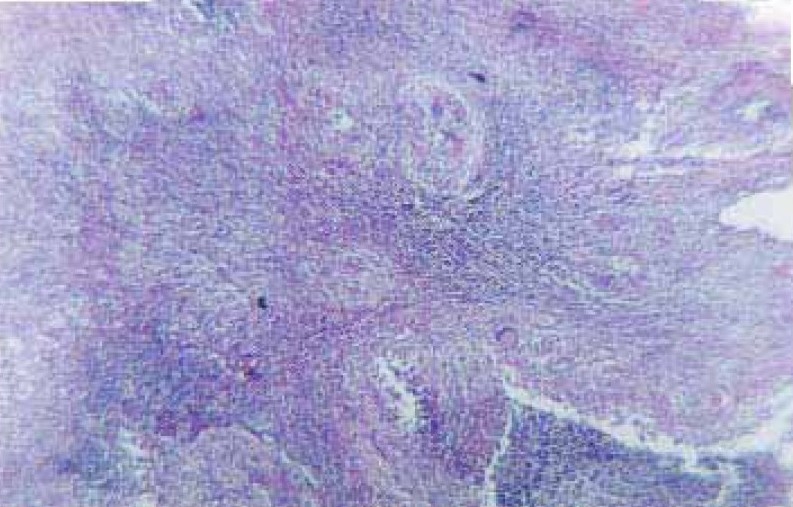
Histopathological examination of the biopsy specimen from splenic lesion showing epitheloid cells granuloma with central necrosis and langhan's giant cells (thick arrow). Lymphoid cells of white pulp of spleen are visible in the slide (thin arrow). (H&E stain; 10X)

Ultrasonography of abdomen showed multiple small hypoechoic foci in the spleen. Computed tomography of the abdomen (Figure 1A–1D) revealed an enlarged spleen that was having multiple small focal hypodense lesions; the appearance was suggestive of abscesses. CT-guided biopsy from the splenic lesions was undertaken. The histopathological examination of the biopsy specimen showed epitheloid cells granuloma with central necrosis along with langhan`s giant cells (Figure 2). Culture of the specimen on Lowenstein Jensen medium isolated Mycobacterium tuberculosis confirming the diagnosis of tuberculosis of the spleen. She was prescribed anti- tubercular treatment (2H_3_R_3_Z_3_E_3_ / 4H_3_ R_3_) and had a favourable outcome with antitubercular chemotherapy.

## DISCUSSION

Splenic enlargement in association with pyrexia of uncertain origin is a clinical sign commonly observed and encountered during the course of various infectious diseases, splenic infarction and malignancies. However, splenic tuberculosis is rare and restricted largely to immunocompromised population. Epidemiological prevalence of splenic tuberculosis is difficult to ascertain as there has been few isolated case reports of the splenic tuberculosis from the different parts of the world. In a large series of 37 cases with focal lesions of the spleen, Joazlina et al found only 4 cases having the tuberculous etiology.^3^ Splenic tuberculosis usually occurs following the haematogenous spread of infection, as a part of disseminated disease, or, occasionally due to contiguous spread of infection. Immunodeficiency is an important risk factor for splenic tuberculosis. The various immunodeficiency conditions identified in these patients include hematologic abnormalities, diabetes mellitus, HIV infection, organ transplantation, and chronic steroid therapy.^3^,^4^

The clinical presentation of splenic abscess is often non-specific, making the diagnosis difficult and is, probably, one of the reasons for a lower prevalence. Splenic abscess should be considered in patients presenting with fever of undetermined origin and abdominal pain; although splenic infarction can have a similar clinical appearance. Lymphoma may also present with fever of unknown origin and pain over spleen suggesting a primary presentation localized to spleen though it may involve multiple sites.^5^ The other features reported in splenic tuberculosis include splenomegaly, leucocytosis and raised erythrocyte sedimentation rate (ESR).

Prior to the advent of ultrasonography and computed tomography (CT), it was very diffficult to make the diagnosis. At present, CT is the preferred imaging modality, as not only does CT reveal the presence of a splenic abnormality but it gives an indication of its nature, the site for possible biopsy or drainage and follow-up after treatment. The characteristic CT features of splenic tuberculosis include solitary / multiple nodular or saccular foci or hypodense areas in the spleen.^6^

Although, sometimes, the patients who have no microbiological pathogen on the culture of splenic specimen are diagnosed by signs and symptoms of abdominal infection along with CT findings suggestive of abscess, the gold standard for diagnosis remains microbiological and histopathological confirmation of the tuberculous lesion in the splenic specimen obtained by fine needle aspiration or biopsy or after splenectomy. The first-line management of the splenic tuberculosis is considered to be anti-tubercular chemotherapy with a significant number of the patients responding to it. Surgery may be appropriate in subjects having rupture of the spleen or if the anti-tubercular treatment fails.

We report this case of splenic tuberculosis in an immunocompetent woman for its rarity and also to highlight the facts that these patients can be diagnosed convincingly and managed by medical treatment effectively.


